# Supplementary material for: Isolation, Characterization, and Safety Evaluation of the Novel Probiotic Strain Lacticaseibacillus paracasei IDCC 3401 via Genomic and Phenotypic Approaches
Source: Microorganisms. 2023 Dec 31;12(1):85. doi: 10.3390/microorganisms12010085 (PMC10821444; doi:10.3390/microorganisms12010085)
Supplement: Supplementary file 1 [file microorganisms-12-00085-s001.zip › microorganisms-2771755-supplementary.pdf]

Supplementary Materials

Table S1. Pathogenic bacteria used in the study.

Strain	Culture condition
Staphylococcus aureus ATCC 25923	TSB medium (BD Difco), 37°C, 24 h, aerobic condition
Enterococcus faecalis ATCC 29212	BHI medium (BD Difco), 37°C, 24 h, aerobic condition
Bacillus cereus ATCC 14579	Nutrient medium (BD Difco), 30°C, 24 h, aerobic condition
Salmonella Typhimurium ATCC 13311	Nutrient medium, 37°C, 24 h, aerobic condition

Table S2. Genomic information of *L. paracasei* IDCC 3401.

	***Lacticaseibacillus paracasei* IDCC 3401**
Identification	*Lacticaseibacillus paracasei*
Genome size (bp)	2,995,875
GC contents (%)	46.59
CDS	2938
ANI value (%)	98.27

Table S3. Functional genes of *L. paracasei* IDCC 3401.

Eggnog	Description	Count	Ratio (%)
J	Translation, ribosomal structure and biogenesis	178	9.3243
A	RNA processing and modification	0	0.0000
K	Transcription	224	11.7339
L	Replication, recombination and repair	303	15.8722
B	Chromatin structure and dynamics	0	0.0000
D	Cell cycle control, cell division, chromosome partitioning	40	2.0953
Y	Nuclear structure	0	0.0000
V	Defense mechanisms	105	5.5003
T	Signal transduction mechanisms	50	2.6192
M	Cell wall/membrane/envelope biogenesis	137	7.1765
N	Cell motility	10	0.5238
Z	Cytoskeleton	0	0.0000
W	Extracellular structures	0	0.0000
U	Intracellular trafficking, secretion, and vesicular transport	40	2.0953
O	Posttranslational modification, protein turnover, chaperones	66	3.4573
C	Energy production and conversion	130	6.8098
G	Carbohydrate transport and metabolism	248	12.9911
E	Amino acid transport and metabolism	224	11.7339
F	Nucleotide transport and metabolism	128	6.7051
H	Coenzyme transport and metabolism	60	3.1430
I	Lipid transport and metabolism	62	3.2478
P	Inorganic ion transport and metabolism	130	6.8098
Q	Secondary metabolites biosynthesis, transport and catabolism	18	0.9429
R	General function prediction only	0	0.0000
S	Function unknown	830	43.4783
Total	-	2,983	100

Table S4. Genes associated with antibiotic resistance.

	Detection
Vancomycin	n.d.
Ampicillin	n.d.
Clindamycin	n.d.
Erythromycin	n.d.
Kanamycin	n.d.

n.d.: not detected.

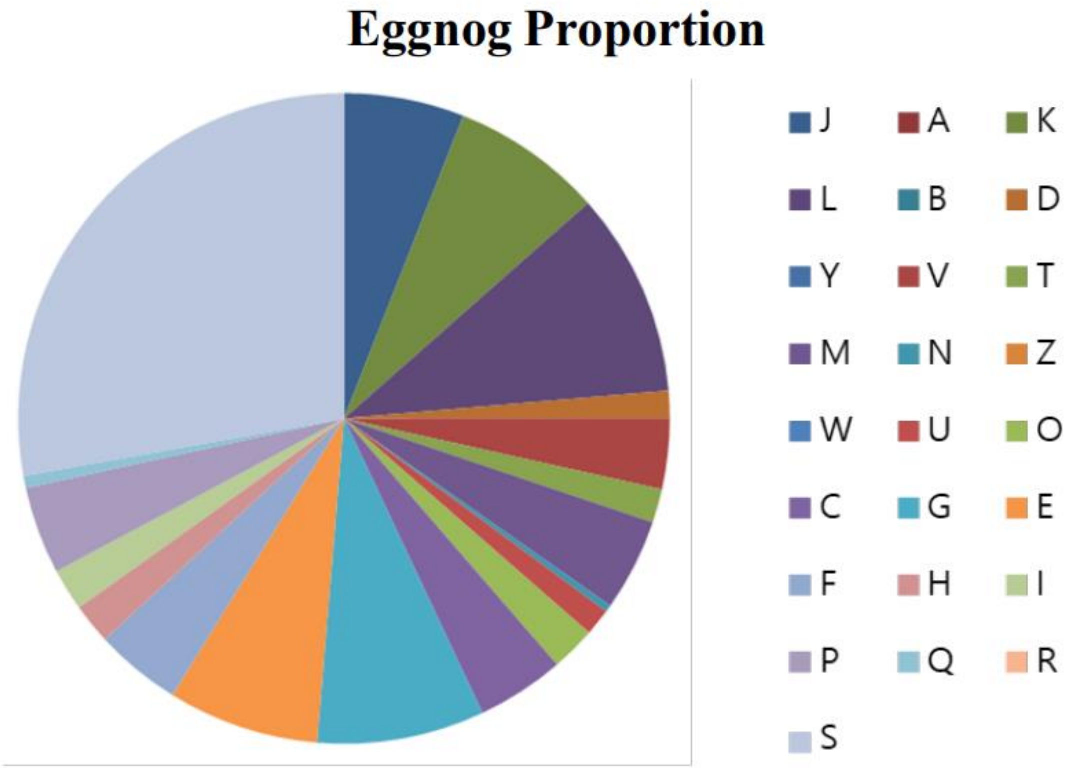

Figure. S1. Functional genes of *L. paracasei* IDCC 3401

Functional genes are analyzed by the eggNOG-mapper v2. J, translation, ribosomal structure and biogenesis; A, RNA processing and modification; K, transcription; L, replication, recombination, and repair; D, cell cycle control, cell division, chromosome partitioning; Y, nuclear structure; V, defense mechanisms; T, signal transduction mechanisms; M, cell wall/membrane/envelope biogenesis; N, cell motility; Z, cytoskeleton; W, extracellular structures; U, intracellular trafficking, secretion, and vesicular transport; O, posttranslational modification, protein turnover, and chaperones; C, energy production and conversion; G, carbohydrate transport and metabolism; E, amino acid transport and metabolism; F, nucleotide transport and metabolism; H, coenzyme transport and metabolism; I, lipid transport and metabolism; P, inorganic ion transport and metabolism; Q, secondary metabolite biosynthesis, transport, and catabolism; R, general function prediction only; S, function unknown.
